# Editorial Melange

**Published:** 1892-02

**Authors:** 


### EDITORIAL MELANGE.

THE American Pharmaceutical Association, together with the state and local pharmacy organizations, are uniting to hold an auxiliary congress during the World's Columbian Exposition in Chicago, in 1893.

The Chairman of the Pharmaceutical Committee, Dr. Oscar Oldberg, has issued a very courteous letter, asking pharmaceutical, chemical and medical journals to assist in giving publicity to the call, and we cheerfully comply with this request. It is to be hoped that a scientific *esprit de corps* will be stimulated by the Columbian Exposition that will pervade every department of applied science.

Dr. Oldberg's committee has issued an elaborate address, which every pharmacist should read.

---

THE Food Manufacturers' Association of the United States proposes to hold a Food Exposition at Madison Square Garden, New York, in October, 1892. This forms a part of a comprehensive plan to celebrate the 400th anniversary of the discovery of America throughout the country, and it is to be commended as one of much merit, and one which will result in much good to the people. Only food products will be allowed on exhibition at this unique fair. It will be the first of the kind ever held in the world. It is hoped that every resident of the State of New York, in particular, will be interested in the exposition, but the people of the whole United States cannot fail to take interest in any attempts to improve the quality of our food, and to elevate and maintain a standard of purity and wholesomeness in regard to our food products.

---

THE COLLEGE OF PHYSICIANS OF PHILADELPHIA announces that the next award of the Alvarenga Prize, being the income for one year of the bequest of the late Senor Alvarenga, and amounting to about one hundred and eighty dollars, will be made on July 14, 1892. Essays intended for competition may be upon any subject in medicine, and must be received by the secretary of the College on or before May 1, 1892. It is a condition of competition that the successful essay, or a copy of it, shall remain in possession of the College.

---

THE NEW YORK POST-GRADUATE HOSPITAL, including the babies' ward, has courteously sent us its seventh annual report, for the year ending September 15, 1891. The members of this corporation embrace some of the most distinguished business men, divines, lawyers, and physicians in the city of New York, and the officers of the hospital are chosen especially for their proficiency. The directors may well take pride in the showing made in this report, both with reference to the conduct of the hospital itself, as well as to its financial condition. We are sure the Post-Graduate will continue in the future, as in the past, to maintain a standard that will command the respect and confidence of the profession throughout the entire Union.

---

THE *Medical Fortnightly*, edited and published by Dr. Bransford Lewis, in St. Louis, Mo., made its appearance with the beginning of the year. It is rarely that a new journal presents such an excellent first number; it teems and bristles with bright sayings of great professional interest, and shows in its dress and make-up, as well as its editorial conduct, that it has an experienced hand at the helm. We bespeak for this handsome magazine a bright and successful future.

---

THE *Southern Medical Record* presents itself in a fine dress to celebrate the beginning of the year 1892, and adds to its editorial staff two of the most celebrated surgeons of the South, Dr. J. McFadden Gaston, and Dr. Willis F. Westmoreland, of Atlanta. These two distinguished teachers and writers will add a tower of strength to this excellent magazine.

The names of Drs. Nicholson and Stockton, who formerly belonged to the editorial staff, have retired from its pages, on account of pressing duties in other directions.

---

THE *Southern Practitioner*, edited by that veteran, Dr. Deering J. Roberts, of Nashville, made its appearance in January with a handsome new dress, and with an enterprising spirit pervading its pages, which indicates that its accomplished and experienced editor does not intend to be outdone by any magazine in the Southwest. We always welcome the *Southern Practitioner* to our table, and we congratulate its editor upon the progressive spirit which he has manifested.

---

MESSRS. WILLIAM WOOD & COMPANY announce that the further publication of the Medical and Surgical Monographs will cease with the issue of the number for December, 1891, but the thirty-six numbers, comprising twelve volumes, can be purchased either in separate numbers, price $1 each, or as bound books, containing three numbers each, as issued. A prospectus containing prices, description of binding, and terms of sale of the bound form of the work, will be sent upon application to the publishers.

Wood's Medical and Surgical Monographs are not supplied through the book trade on any terms. All orders should be sent direct to William Wood & Company, Medical Publishers, New York.

---

A UNIQUE MAGAZINE.—A unique experiment will be tried in the February issue of *The Ladies' Home Journal*. The entire number has been contributed in prose, fiction and verse, by the daughters of famous parentage, as a proof that genius is often hereditary. The work of thirty of these "daughters" will be represented. These will comprise the daughters of Thackeray, Hawthorne, Dickens, James Fennimore Cooper, Horace Greeley, Mr. Gladstone, President Harrison, William Dean Howells, Senator Ingalls, Dean Bradley of Westminster, Julia Ward Howe, General Sherman, Jefferson Davis, and nearly a score of others. Each article, poem or story, printed in this number has been especially written for it, and the whole promises to be a successful result of an idea never before attempted in a magazine.

---

ANNOUNCEMENT.—E. B. Treat, Publisher, New York, has in press for early publication the 1892 International Medical Annual, being the tenth yearly issue of this deservedly popular work.

Its corps of thirty-five editors are specialists in their respective departments, and have been carefully selected from the brightest and best American, English, and French authors.

It is the embodiment of what is worth preserving of the current medical journals of the world for the year, and will contain over 6,000 references to diseases and their remedies.

The service rendered the profession by this Annual cannot be overestimated, and it is an absolute necessity to every physician who would keep abreast with the continuous progress of practical medical knowledge.

This Index of New Remedies and Dictionary of New Treatment, epitomized in one ready reference volume at the low price of $2.75, makes a desirable investment for the busy practitioner, student and chemist.

---

Dr. W. E. B. Davis, late of Birmingham, Ala., has removed to Rome, Ga., to join his forces with Dr. J. B. S. Holmes in the conduct of the largest and most completely equipped sanitarium in the South, devoted exclusively to the treatment of diseases of women. The temptation must have been a very strong one to induce Dr. Davis to abandon his large and lucrative practice in Birmingham, but we have no doubt that he will find in his new home, and in the new relations which he assumes, a more congenial field, where he can cultivate his peculiar fitness and taste for the practice of abdominal surgery to its fullest extent. Dr. Davis is easily and properly regarded as the foremost abdominal surgeon in the mid-south, and is also one of the most popular and progressive physicians of the age. His ideal is a high one, and it would benefit the whole profession if it should follow Dr. Davis's standard and aim to reach its high pinnacle.

---

The Transactions of the American Association of Obstetricians and Gynecologists, Vol. IV., 1891, is now ready for delivery. It is a handsome standard octavo, bound in half Russia and cloth, similar to the preceding numbers, is handsomely illustrated, and in every way maintains the high standard of its predecessors. Price, cloth, $5.00; half Russia, $6.00. The edition is limited, and orders should be placed with the undersigned at once, as it will not be possible to obtain the volume a few months hence.

WILLIAM WARREN POTTER, M.D., *Sec'y*,
284 Franklin street, Buffalo, N. Y.

---

The Medical Society of the State of New York will hold its eighty-sixth annual meeting at the City Hall, in Albany, Tuesday,

Wednesday, and Thursday, February 2d, 3d, and 4th, under the presidency of Dr. A. Walter Suiter, of Herkimer. The annual banquet of the Society will take place at the Delavan House, Wednesday evening, February 3d.

It is anticipated that this will be one of the largest meetings the Society has ever held, and it is requisite that those who expect to attend shall be expeditious in securing their hotel accommodations. A number of distinguished gentlemen from outside the State will be present to add to the interest of the occasion.

---

THE restraining by statutory law of the commerce in poisonous drugs, such as opium, cocaine and chloral, is *correct* in principle, and should receive the endorsement of every citizen who has respect for human life. We hope to see a bill passed in our state legislature that will prevent the sale of such drugs except upon the prescription of a physician.

---

DR. FERDINAND KING, of New York, has severed his relations with the *International Journal of Surgery*, and established the *Doctors' Weekly*, which is a strictly medical newspaper. It is a bright and handsome sheet that deserves to succeed. It has our best wishes, at any rate, whatever they may be worth. It is published at 33-39 Gold street, New York, where subscriptions should be sent, addressed to Dr. King.

---

THE HARVARD MEDICAL SCHOOL ASSOCIATION has published its Bulletin No. 1, which comprises a report of the first annual meeting, held in Boston, June 23, 1891. This is a handsomely printed book of fifty-two pages that every alumnus of a school should possess, and if there are any who have not received it, they can obtain it by sending their names to Dr. R. W. Lovett, Secretary, No. 379 Boylston street, Boston, Mass.

